# Metformin Promotes Mechanical Stretch-Induced Skin Regeneration by Improving the Proliferative Activity of Skin-Derived Stem Cells

**DOI:** 10.3389/fmed.2022.813917

**Published:** 2022-05-24

**Authors:** Shaoheng Xiong, Wei Liu, Yajuan Song, Jing Du, Tong Wang, Yu Zhang, Zhaosong Huang, Qiang He, Chen Dong, Zhou Yu, Xianjie Ma

**Affiliations:** Department of Plastic Surgery, Xijing Hospital, Fourth Military Medical University, Xi’an, China

**Keywords:** skin expansion, metformin, mechanical stretch, skin regeneration, hair follicle bulge stem cells, epidermal stem cells

## Abstract

**Background:**

Skin expansion by mechanical stretch is an essential and widely used treatment for tissue defects in plastic and reconstructive surgery; however, the regenerative capacity of mechanically stretched skin limits clinical treatment results. Here, we propose a strategy to enhance the regenerative ability of mechanically stretched skin by topical application of metformin.

**Methods:**

We established a mechanically stretched scalp model in male rats (*n* = 20), followed by their random division into two groups: metformin-treated (*n* = 10) and control (*n* = 10) groups. We measured skin thickness, collagen volume fraction, cell proliferation, and angiogenesis to analyze the effects of topical metformin on mechanically stretched skin, and immunofluorescence staining was performed to determine the contents of epidermal stem cells and hair follicle bulge stem cells in mechanically stretched skin. Western blot was performed to detect the protein expression of skin-derived stem cell markers.

**Results:**

Compared with the control group, metformin treatment was beneficial to mechanical stretch-induced skin regeneration by increasing the thicknesses of epidermis (57.27 ± 10.24 vs. 31.07 ± 9.06 μm, *p* < 0.01) and dermis (620.2 ± 86.17 vs. 402.1 ± 22.46 μm, *p* < 0.01), number of blood vessels (38.30 ± 6.90 vs. 17.00 ± 3.10, *p* < 0.01), dermal collagen volume fraction (60.48 ± 4.47% vs. 41.28 ± 4.14%, *p* < 0.01), and number of PCNA+, Aurora B+, and pH3+ cells. Additionally, we observed significant elevations in the number of proliferating hair follicle bulge stem cells [cytokeratin (CK)15+/proliferating cell nuclear antigen (PCNA)+] (193.40 ± 35.31 vs. 98.25 ± 23.47, *p* < 0.01) and epidermal stem cells (CK14+/PCNA+) (83.00 ± 2.38 vs. 36.38 ± 8.96, *p* < 0.01) in the metformin-treated group, and western blot results confirmed significant increases in CK14 and CK15 expression following metformin treatment.

**Conclusion:**

Topical application of metformin enhanced the regenerative capacity of mechanically stretched skin, with the underlying mechanism possibly attributed to improvements in the proliferative activity of skin-derived stem cells.

## Introduction

Skin expansion is a viable method for mechanical stretch-induced skin regeneration (1). In the clinic, skin expansion is generally applied in skin-defect repair and organ reconstruction by plastic surgeons and represents a feasible way to achieve clinically complete skin regeneration *in vivo* by mechanical stretching. However, the regenerative capacity of mechanically stretched skin is limited (2). Thus, there is an immediate unmet need to explore novel approaches to promote skin regeneration for better clinical applications.

Multiple biological behaviors, including cell proliferation, dermal collagen synthesis, and angiogenesis, influence skin regeneration during the mechanical stretching process. Previous studies show that stem cell therapy using bone marrow-derived stem cells and/or adipose-derived stem cells promotes mechanically stretched skin regeneration (2–5). However, skin-derived stem cells, especially epidermal stem cells (ESCs) and hair follicle bulge stem cells (HFBSCs) (6), might also perform critical regenerative functions during skin expansion. Therefore, in this study, we mainly focused on the biological effects of ESCs and HFBSCs during mechanical stretching. Our previous work demonstrated that HFBSC transplantation enhanced skin regeneration during the mechanical stretch process by differentiation into epidermal cells, vascular endothelial cells, and the outer root sheath cells of hair follicles (7). However, the direct application of stem cells *in vitro* in clinical practice is difficult. Thus, identifying a pharmacotherapy capable of positively regulating these above biological behaviors and activating ESCs or HFBSCs might contribute to the regeneration of mechanically stretched skin.

Metformin is a first-line medication used to treat type 2 diabetes, as recommended by the American Diabetes Association (8). Growing evidence shows that topical application of metformin might favor tissue regeneration and wound repair, which partially correlates with promoting angiogenesis and activating HFBSCs (9, 10). Other studies suggest that metformin accelerates wound healing by increasing dermal collagen deposition and re-epithelialization (10–14). Coincidentally, the mechanical stretching process involves both cyclic damage and repair. These findings suggest that the topical application of metformin might represent an alternative option for improving mechanical stretch-induced skin regeneration.

In this study, we investigated the ability of metformin to promote regeneration of mechanically stretched skin. To test this hypothesis, we constructed a rat scalp-expansion model to observe the effects of metformin on mechanically stretched skin regeneration and focused on the changes in ESCs and HFBSCs.

## Materials and Methods

### Animals and the Mechanical Stretch Model

All animal procedures were performed according to protocols approved by the Institutional Animal Care and Use Committee of the Fourth Military Medical University (approval no. IACUC-20120117). Male Sprague–Dawley rats (6-week old; 180–220 g) were purchased from the Animal Center of the Fourth Military Medical University (Xi’an, China). Twenty rats were used in total, with 10 in the metformin-treated group and 10 in the control group. Rats were housed in groups of five animals per cage and *ad libitum* access to food and water in a 12-/12-h light/dark cycle.

**FIGURE 1:**
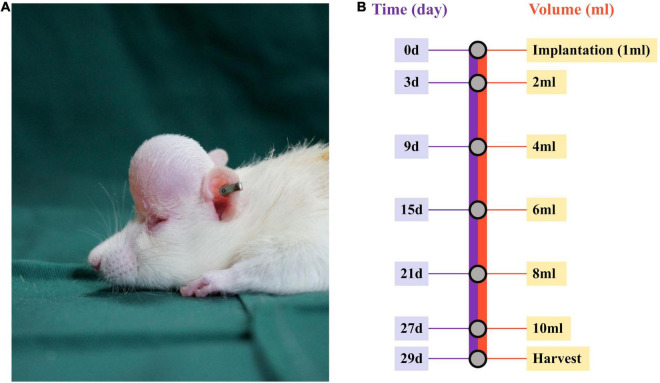
Establishment of the mechanical stretch model and experimental design. **(A)** A round silicone expander was implanted under the rat scalp, and **(B)** 1 mL of normal saline was injected into the expander every other day up to a final injection on day 27. At 48 h after the final injection, the mechanically stretched skin was harvested for analysis.

A mechanical stretch model was established, as described previously (15). Briefly, rats were placed in the prone position after being anesthetized (1.5% pentobarbital sodium; 40 mg/kg body weight), and the neck skin of each rat was then incised at ∼1.5 cm, followed by implantation of a customized round 1-mL silicone expander (Guangzhou Wanhe Plastic Materials Co., Ltd., Guangzhou, China) under the scalp after blunt dissection. Normal saline (1 mL) was injected every 2 days to a final injection volume of 10 mL (Figure 1). From the beginning of the expansion, rats were treated topically every other day with 300 mM metformin (Sigma-Aldrich, St. Louis, MO, United States) or vehicle (double-steamed water). The animals were subjected to sample collection on day 29 and immediately euthanized (Figure 1B).

### Skin Microvascular Flow Analysis

Analysis of microvascular flow in mechanically stretched skin was performed, as described previously (7). Dermal microvascular blood-flow intensity was measured using laser Doppler flowmetry (moorFLPI Full-Field Laser Perfusion Imager 3.0; Moor Instruments Ltd., Axminster, United Kingdom). The mean value of 10 independent images from each rat was used to represent blood flow. After measuring and collecting blood-flow images of the mechanically stretched skin, data were analyzed using onboard software (MoorFLPI Image Review v.2.0; Moor Instruments Ltd., Axminster, United Kingdom).

### Histologic Analysis

Tissue samples were fixed in 4% paraformaldehyde (Sigma-Aldrich, St. Louis, MO, United States) in phosphate-buffered saline (PBS) for 24 h and embedded in paraffin, after which 4-μm-thick sections were stained with hematoxylin and eosin (H&E; Servicebio, Wuhan, China), Picrosirius Red (Servicebio), and Masson’s trichrome (Servicebio) for conventional morphological evaluation after deparaffinization and rehydration. For the measurement of epidermal and dermal thickness, five fields from each section were randomly selected for statistical analysis. Collagen fibers were observed using polarized light microscopy (Eclipse Ci; Nikon, Tokyo, Japan), with collagen I fibers stained red and newly synthesized fibers stained yellow to orange (16), whereas collagen III fibers appeared green. Collagen volume fraction (CVF) was used to assess the relative content of collagen in the dermis stained with Masson’s trichrome.

### Immunohistochemical and Immunofluorescence Staining

Tissue specimens embedded in paraffin were cut into 4-μm-thick sections and subjected to antigen retrieval at 96°C for 20 min in citrate buffer (pH 6.0), followed by blocking for 1 h at room temperature with 5% goat serum in PBS. The following antibodies were used to investigate protein expression via IHC analysis: anti-Ki67 (1:500; Cell Signaling Technology, Danvers, MA, United States), anti-proliferating cell nuclear antigen (PCNA; 1:1000; Abcam, Cambridge, United Kingdom), anti-Aurora B (1:150; Abcam), anti-histone H3 (phospho S10; pH3; 1:600; Abcam), and anti-CD31 (1:2000; Cell Signaling Technology, Danvers, MA, United States) primary antibodies and a horseradish peroxidase-conjugated goat anti-rabbit secondary antibody (Jackson Laboratories, Bar Harbor, ME, United States). For immunofluorescence staining, the following primary antibodies were applied overnight at 4°C in a humidified chamber: mouse anti-rat PCNA (1:500; Abcam), rabbit anti-rat cytokeratin (CK)14 (1:200; Proteintech, Wuhan, China), and rabbit anti-rat CK15 (1:400; Proteintech). After washing in PBS, the sections were incubated with Alexa Fluor 488-conjugated goat anti-mouse IgG and IgM (H + L) secondary antibodies (1:1000; Invitrogen, Carlsbad, CA, United States) and an Alexa Fluor 568-conjugated goat anti-rabbit IgG (H + L) cross-adsorbed secondary antibody (1:1000; Invitrogen) for 1 h at room temperature in a humidified chamber. Nuclei were counterstained with 10 μg/mL 4′,6-diamidino-2-phenylindole (Invitrogen) for 15 min. Laser scanning confocal microscopy was performed using a confocal microscope (Nikon). Positive cell counting based on Ki67, PCNA, Aurora B, and pH3 was utilized to detect proliferating cells using ImageJ software (v.1.53k; NIH, Bethesda, MD, United States), and microvessel density (MVD) was determined by calculating the number of CD34+ blood vessels per field.

### Transmission Electron Microscopy

Samples used for TEM were prepared, as described previously (17). The harvested skin sample was cut into small pieces (5 mm^3^) and fixed in 2.5% glutaraldehyde (Sigma-Aldrich, St. Louis, MO, United States) in 0.1 M PBS for 24 h at 4°C. The samples were then post-fixed with 1% osmium tetroxide solution for 12 h, dehydrated with a descending ethanol series for 2 h, and embedded in epon resin. The area representative of the dermis was retained for ultrastructural analysis. Subsequently, 80-nm-thick ultrathin sections were cut and placed on copper grids for observation under a JEM-1400 transmission electron microscope (JEOL Ltd., Tokyo, Japan).

### Western Blot Analysis

After homogenization, all skin tissues were lysed for 30 min with ice-cold radioimmunoprecipitation assay lysis buffer (CoWin Biosciences, Taizhou, China). Protein lysate (15 μg; concentration determined by a BCA assay) was subjected to sodium dodecyl sulfate polyacrylamide gel electrophoresis (SDS-PAGE; CoWin Biosciences, Taizhou, China) on a 6% gel for collagen I and collagen III; 10% gel for Aurora B, CK14, and CK15; 12% gel for PCNA, pH3, and glyceraldehyde 3-phosphate dehydrogenase (GAPDH) at 80 V for 1.5 h, and then transferred to polyvinylidene fluoride membranes (Merck, Darmstadt, Germany). The membranes were blocked with 5% bovine serum albumin in PBS at room temperature for 1.5 h, followed by incubation with primary antibodies against collagen I (1:1000; Cell Signaling Technology, Danvers, MA, United States), collagen III (1:1000; Cell Signaling Technology, Danvers, MA, United States), PCNA (1:1000; Abcam), Aurora B (1:1000; Abcam), pH3 (1:200; Abcam), CK14 (1:1000; Proteintech), CK15 (1:2000; Proteintech), and GAPDH (1:20,000; Proteintech) at 4°C overnight. Quantitative analysis was performed on the immunoreactive bands using ImageJ software (v.1.53k; NIH, Bethesda, MD, United States).

### Statistical Analysis

Data are expressed as the mean ± standard deviation. Student’s *t*-test and a Mann–Whitney non-parametric test were performed to compare the data of unpaired samples using GraphPad Prism software (v.8.0; GraphPad Software, La Jolla, CA, United States). A *p* < 0.05 was considered significant.

## Results

### Metformin Enhances Skin Thickening After Mechanical Stretching

**FIGURE 2:**
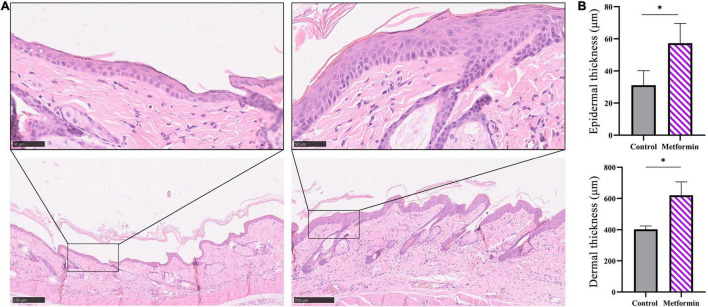
Measurement of the thickness of mechanically stretched skin. **(A)** H&E staining of skin from the metformin-treated and control groups. Compared with the control group (top left), the metformin-treated group had more cell layers in the epidermis, with the cells closely arranged in the epidermal stratum basale layer (top right) and numerous hair follicles present in the metformin-treated group (bottom right). **(B)** Quantification of epidermal and dermal thickness. The epidermis and dermis in the metformin-treated group were significantly thicker than those in the control group. *p < 0.05.

Hematoxylin and eosin staining images showed an increased number of cell layers in the epidermis of the metformin-treated group, with the cells closely arranged in the epidermal stratum basale layer. Additionally, we observed numerous hair follicles in the dermis of mechanically stretched skin in the metformin-treated group. Moreover, statistical analysis confirmed that the thickness of mechanically stretched skin increased following metformin treatment (Figures 2A,B). Furthermore, mechanically stretched epidermis and dermis were thicker in the metformin-treated group relative to that in the control group [57.27 ± 10.24 vs. 31.07 ± 9.06 μm (*p* < 0.01) and 620.2 ± 86.17 vs. 402.1 ± 22.46 μm (*p* < 0.01), respectively] (Figure 2B).

### Metformin Increases Dermal Collagen Deposition and Arrangement

**FIGURE 3:**
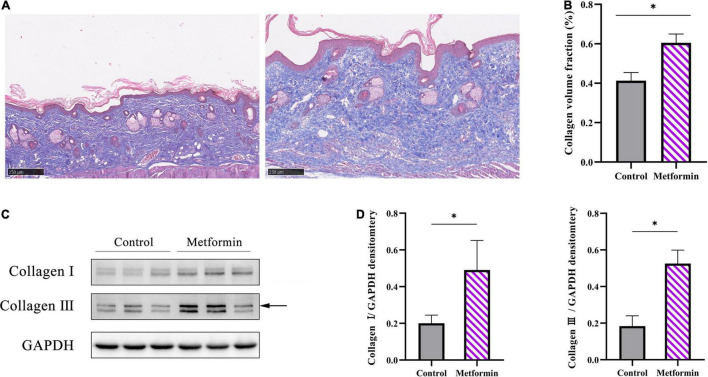
Evaluation of collagen synthesis. **(A)** Masson’s trichrome staining showed increased collagen deposition in the metformin-treated group, with **(B)** a significantly higher CVF than the control group. **(C)** Western blot analysis and **(D)** quantification of collagen I and collagen III expressions. *p < 0.05.

**FIGURE 4:**
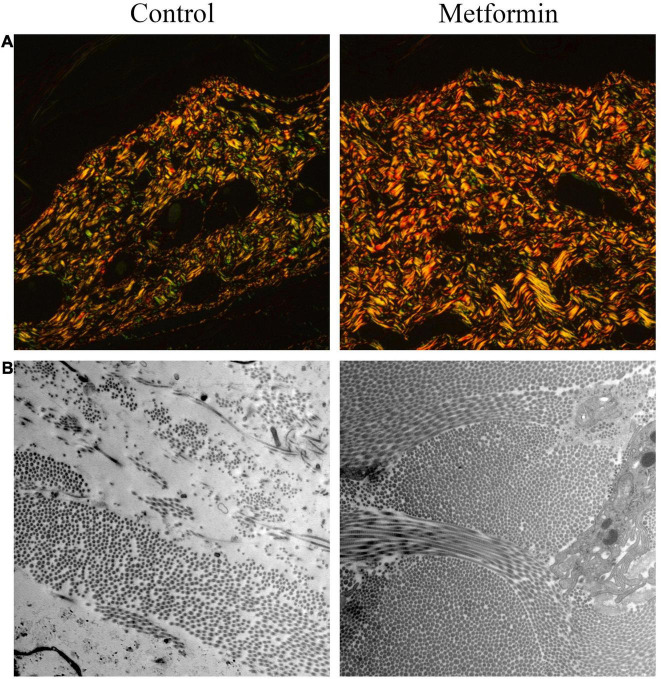
Picrosirius Red staining ultrastructural analysis of dermal collagen fibrils. **(A)** Picrosirius Red images showed increased neocollagen development (yellow to orange) in the metformin-treated group. **(B)** TEM images revealed an improved arrangement of collagen fibrils in the metformin-treated group without a distorted ultrastructure, whereas collagen fibrils in the control group were broken and disorganized. Magnification: 15,000×.

Masson’s trichrome staining to determine collagen deposition in the mechanically stretched dermis (Figure 3A) revealed that the metformin-treated group showed a higher CVF than the control group (60.48 ± 4.47% vs. 41.28 ± 4.14%, *p* < 0.01) (Figure 3B). Additionally, western blot results confirmed significant increases in collagen I and collagen III expression in the metformin-treated group (Figures 3C,D), and Picrosirius Red staining revealed more newly synthesized collagen in the metformin-treated group (Figure 4A). Furthermore, ultrastructural analysis showed more evenly arranged collagen fibrils without distortion in the metformin-treated group, whereas more broken and disorganized collagen fibrils were observed in the control group (Figure 4B).

### Metformin Increases Cell Proliferation

**FIGURE 5:**
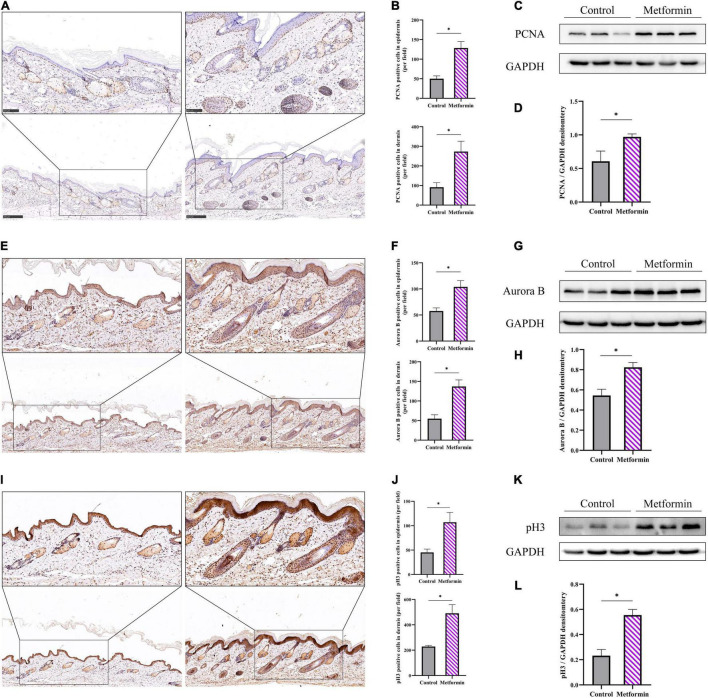
Cell proliferation in the mechanically stretched skin. Immunohistochemistry (IHC) staining of PCNA **(A)**, Aurora B **(E)**, and pH3 **(I)** in mechanically stretched skin. Proliferative cells were mainly located in the basal layer of the epidermis and near the dermal hair follicles. More proliferating PCNA+ **(B)**, Aurora B+ **(F)**, and pH3+ **(J)** cells were present in both the epidermis and dermis of metformin-treated skin. Western blotting was used to detect the protein expression of PCNA **(C)**, Aurora B **(G)**, and pH3 **(K)**, and increased expression of PCNA **(D)**, Aurora B **(H)**, and pH3 **(L)** was observed in the metformin-treated group. *p < 0.05.

The metformin-treated group showed significantly increased proportions of Ki67+ cells per field as compared with the control group (epidermis: 25.25 ± 1.71 vs. 76.00 ± 12.44, *p* < 0.01; dermis: 72.75 ± 4.03 vs. 143.00 ± 27.76, *p* < 0.01; Supplementary Figures 1A,B). Additionally, we respectively analyzed IHC staining of PCNA (Figures 5A,B), Aurora B (Figures 5E,F), and pH3 (Figures 5I,J) as markers for cell proliferation in different phases of the cell division cycle. We found more proliferating cells (PCNA+, Aurora B+, or pH3+) in the metformin-treated group than in the control group [epidermis: PCNA+ cells (50.25 ± 7.93 vs. 128.5 ± 16.60, *p* < 0.01), Aurora B+ cells (57.60 ± 5.94 vs. 103.9 ± 11.94, *p* < 0.01), and pH3+ cells (45.20 ± 6.87 vs. 107.00 ± 20.13, *p* < 0.01); dermis: PCNA+ cells (91.00 ± 23.08 vs. 273.3 ± 52.99, *p* < 0.05), Aurora B+ cells (54.75 ± 10.05 vs. 137.00 ± 16.70, *p* < 0.01), and pH3+ cells (228.50 ± 8.55 vs. 490.70 ± 68.49, *p* < 0.01); Figures 5B,F,J]. These proliferative cells were mainly located in the basal layer of the epidermis and the outer root sheath of hair follicles. Furthermore, western blot results confirmed that the protein level of PCNA (Figures 5C,D), Aurora B (Figures 5G,H), and pH3 (Figures 5K,L) were all markedly increased in the metformin-treated group.

### Metformin Increases Blood Flow and the Number of Blood Vessels in the Skin

**FIGURE 6:**
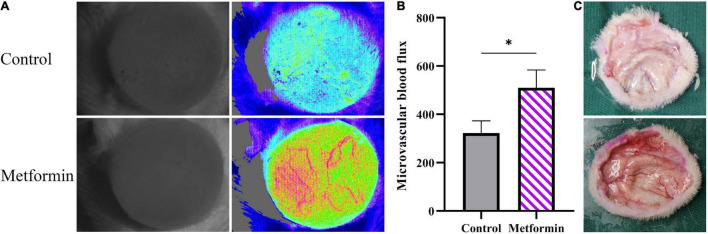
Detection of microcirculatory blood flow. **(A)** Microcirculatory blood flows analysis of mechanically stretched skin from the metformin-treated and control groups. **(B)** The results showed significantly increased blood flow in the metformin-treated group and **(C)** a higher number of blood vessels observed in freshly isolated skin from the metformin-treated group relative to the control group. *p < 0.05.

**FIGURE 7:**
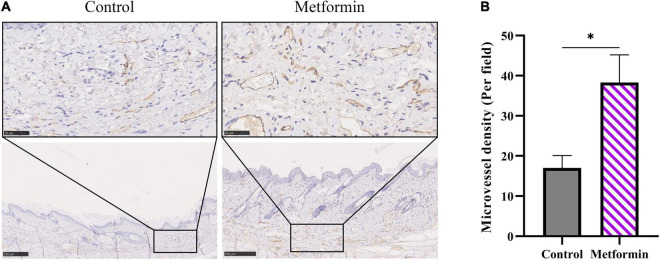
IHC staining of CD31 in mechanically stretched skin. **(A)** IHC staining revealed more CD31+ vessels in mechanically stretched skin from the metformin-treated group relative to the control group. **(B)** Quantitative analysis of microvascular density. *p < 0.05.

*In vivo* detection of microvascular blood flow showed improved blood flux in the metformin-treated group than in the control group (510.00 ± 73.59 vs. 322.40 ± 50.60, *p* < 0.05) (Figures 6A,B). Additionally, we identified an increased number of blood vessels in the mechanically stretched skin grafts (Figure 6C), with IHC staining of CD31 revealing more blood vessels per field in the metformin-treated group relative to the control group (38.30 ± 6.90 vs. 17.00 ± 3.10, *p* < 0.01) (Figures 7A,B).

### Metformin Increases the Proliferative Capability of Skin-Derived Stem Cells

**FIGURE 8:**
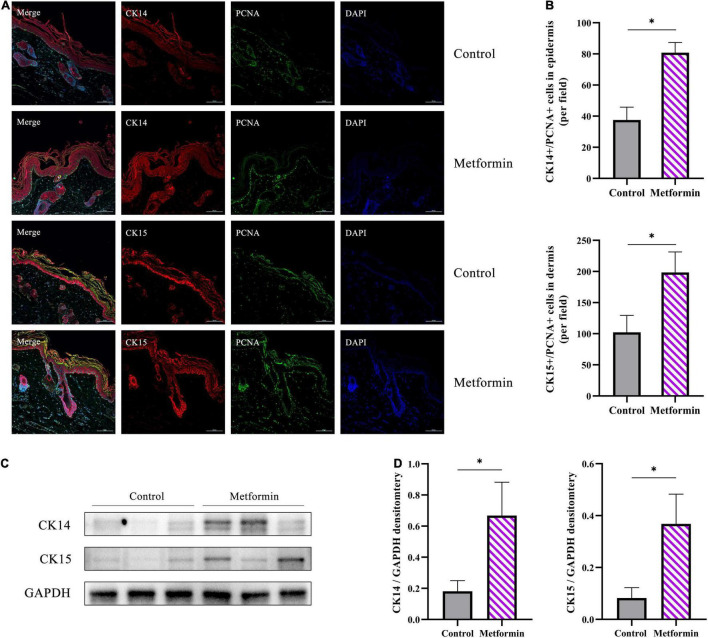
Immunofluorescence staining of mechanically stretched skin. **(A)** Double-immunofluorescence staining of PCNA (green) and CK14 (red) or CK15 (red) revealed the proliferative status of epidermal stem cells (ESCs) and hair follicle bulge stem cells (HFBSCs). More CK14+/PCNA+ ESCs and CK15+/PCNA+ HFBSCs were identified in mechanically stretched skin from the metformin-treated group than in the control group. **(B)** Quantitative analysis revealed significantly increased numbers of CK14+/PCNA+ and CK15+/PCNA+ cells in skin samples from the metformin-treated group. **(C)** Western blot analysis and **(D)** quantification of CK14 and CK15 protein expression. The results showed that the protein levels of CK14 and CK15 were significantly increased in the metformin-treated group relative to those in the control group. *p < 0.05.

To determine the types of cells involved in skin regeneration, we performed double-immunofluorescence staining of PCNA and CK14 or CK15. The results showed that metformin treatment significantly increased the number of CK14+/PCNA+ cells in the stratum basale layer of the mechanically stretched epidermis (83.00 ± 2.38 vs. 36.38 ± 8.96, *p* < 0.01), with proliferating hair follicle outer-sheath cells (CK15+/PCNA+) also observed in the metformin-treated group (193.40 ± 35.31 vs. 98.25 ± 23.47, *p* < 0.01; Figures 8A,B). Moreover, western blot results confirmed the upregulated expression of CK14 and CK15 in the metformin-treated group, which was consistent with the results of double-immunofluorescence staining (Figures 8C,D).

## Discussion

Soft-tissue expansion by mechanical stretching represents an efficient surgical procedure for skin-defect repair and organ reconstruction; however, the skin-regenerative capacity is limited (2). Stem cell therapy represents an alternative method for enhancing the regeneration of mechanically stretched skin (2–5). In particular, ESCs and HFBSCs located *in situ* play a vital role in skin regeneration and repair. In this study, we found that metformin enhanced the regenerative capacity of mechanically stretched skin by thickening both the epidermis and dermis, increasing angiogenesis, and promoting cell proliferation in mechanically stretched skin, possibly as a result of the improved proliferative activity of ESCs and HFBSCs.

Angiogenesis and remodeling are important for the regeneration of mechanically stretched skin (18). In the present study, we observed significantly increased *in vivo* microvascular blood flow in mechanically stretched skin following metformin treatment. Moreover, metformin treatment improved the blood perfusion of the mechanically stretched skin, with subsequent histologic evaluation and quantitative assessment of CD31+ blood vessels confirming the improved angiogenesis in the metformin-treated group. Previous studies report that metformin promotes tissue repair directly or indirectly (14), mainly by improving the angiogenesis of wounds (10, 19, 20). Because mechanically stretched skin experiences similar stress and relaxation activities to that in injury- and repair-related mechanical stretching processes (18), the proangiogenic effect of metformin during wound healing is likely replicated in mechanically stretched skin. Previous reports demonstrated that metformin could promote angiogenesis both *in vitro* and *in vivo*. Local application of metformin significantly accelerated wound healing, which was attributed to the promotion of angiogenesis via stimulation of the AMP-activated protein kinase pathway (AMPK) (10). Additionally, metformin promoted angiogenesis and improved the survival of random pattern skin flaps by activating the AMPK–mammalian target of rapamycin–transcription factor EB signaling pathway (20). Moreover, low-dose metformin significantly enhanced the angiogenic differentiation of mesenchymal stem cells *in vivo* and *in vitro* and boosted mesenchymal stem cell-mediated promotion of HUVEC migration and tube formation by upregulating the expression of stem cell factor and vascular endothelial growth factor receptor 2 (21).

The synthesis and deposition of dermal collagen could be advantageous for skin regeneration during mechanical stretching. The present results showed that collagen deposition was significantly increased by metformin treatment and that the ultrastructure of collagen fibers was highly ordered according to TEM analysis. Moreover, we confirmed elevated levels of both collagen I and collagen III expression in the metformin-treated group. Additionally, the promotion of dermal angiogenesis can benefit collagen synthesis by increasing blood supply after treatment with metformin. A previous study reported that chronic topical administration of metformin (once daily for 14 days) accelerated wound healing by promoting collagen deposition (10).

Epidermal thickening is another important characteristic of regenerated mechanically stretched skin. In this study, we found that epidermal thickness was significantly increased in the metformin-treated group, which was mainly attributed to the increased proliferation of epidermal cells. We measured IHC staining to enumerate Ki67+ (cell cycle entry marker), PCNA+ (peak level reached around the late G1-phase and early S-phase of the cell cycle), Aurora B+ (peak level reached around mid-late M-phase of the cell cycle), and pH3+ (peak level reached in the late G2- and M-phase of the cell cycle) cells per field to evaluate cell proliferation in mechanically stretched skin. The results showed that those positive cells were all increased in the metformin-treated group, and subsequent western blotting confirmed that the PCNA, Aurora B, and pH3 protein expression levels were also increased in the metformin-treated group compared to those in the control group. Moreover, IHC staining evaluation revealed that the proliferating cells were primarily located in the stratum basale layer, which probably comprises ESCs. To verify the source of proliferating cells in the stratum basale layer, we further used double-immunofluorescence staining of CK14+/PCNA+ to label ESCs. And the double-immunofluorescence staining showed that numerous CK14+/PCNA+ ESCs were present in the metformin-treated group, which would subsequently drive stronger proliferative activity in the epidermis. Further study confirmed that the expression of CK14 was significantly elevated in the metformin-treated group. Moreover, a recent study revealed that the proliferation and differentiation potential of CK14+ ESCs significantly contributed to the regeneration of mechanically stretched skin (1). Additionally, others found that topical application of metformin benefited re-epithelialization during wound healing (10). These results indicate that metformin treatment might promote epidermal thickening by increasing ESC proliferation and differentiation.

Similarly, we analyzed dermal cell proliferation by IHC staining of PCNA, Aurora B, and pH3. Notably, we found that these positive cells (PCNA, Aurora B, and pH3) were significantly increased in the metformin-treated group compared with numbers in the control group, and most of these positive cells were located in the bulge region, whereas the outer-root sheath of hair follicles was thought to be the site of ESCs and HFBSCs. We next used double-immunofluorescence labeling of CK15+/PCNA+ HFBSCs to confirm the sources of these proliferating cells. We observed a significantly increasing number of CK15+/PCNA+ HFBSCs in mechanically stretched skin following metformin treatment. Further western blot analysis revealed increased CK15 expression in the metformin-treated group, indicating that more HFBSCs were present in metformin-treated mechanically stretched skin than in the control group. Our previous study showed that HFBSC transplantation promoted the regeneration of mechanically stretched skin via direct differentiation into epidermal cells, vascular endothelial cells, and the outer-root sheath cells of hair follicles (7). Therefore, we suggest that topical application of metformin might improve the regenerative capacity of mechanically stretched skin by activating local HFBSCs. Previous studies reported a higher number of hair follicles present at the wound edge following metformin treatment along with more rapid wound healing (10). Furthermore, previous studies showed that HFBSC activation is involved in skin regeneration and re-epithelialization during wound repair (22). These results suggest that metformin might benefit wound healing by activating HFBSCs present in hair follicles. Growing evidence suggests that metformin exerts pleiotropic effects to activate stem cells and promote functional rejuvenation (9, 23–28). Among these effects, topical application of metformin reactivated dormant HFBSCs, which subsequently contributed to initiating new anagen hair growth (9). Notably, a clinical randomized controlled trial comparing therapeutic efficacy between hair-follicle scalp grafts and non-hairy skin grafts demonstrated that transplanted skin with more hair follicles results in better wound healing (29). This evidence supports our finding that topical application of metformin further activated the proliferative capacity of HFBSCs.

Previous studies have demonstrated the effect of metformin on the hair follicle; however, there was no direct evidence that metformin promotes the proliferation of ESCs or HFBSCs. These mainly focused on evaluating hair growth and the number of hair follicles. In this study, we provided direct evidence that the topical application of metformin could promote the proliferation of ESCs and HFBSCs using IHC staining, double-immunofluorescence staining, and measurement of protein expression levels. In addition, previous work mainly focused on applying metformin to promote wound healing. Nevertheless, there currently is no evidence to confirm the effect of the topical application of metformin on mechanical stretch-induced skin regeneration. Skin expansion is an important means to clinically obtain complete skin regeneration induced by mechanical stretching *in vivo*. Although previous studies have shown that stem cells could effectively promote expanded skin regeneration, alternative methods are required due to clinical ethical restrictions. This study found that HFBSCs activated by locally applied metformin might directly participate in the skin-regeneration process during mechanical stretching. These findings offer novel insights into improving mechanical stretch-induced skin regeneration by activating local HFBSCs.

However, this study has two limitations. First, the skin anatomy is not identical between rats and humans; therefore, species-specific differences in the observed therapeutic effects likely exist. Further clinical evidence in humans is needed to confirm these findings. Second, metformin’s mechanisms associated with proliferative regulation of skin-derived stem cells, including HFBSCs and ESCs, are complex and require further investigation.

## Conclusion

In summary, we found that topical application of metformin promoted the regenerative capacity of mechanically stretched skin, possibly attributed to enhanced collagen synthesis, angiogenesis, and skin-derived stem cell proliferation. These findings might provide a valuable alternative strategy for promoting mechanical stretch-induced skin regeneration.

## Data Availability Statement

The original contributions presented in the study are included in the article/Supplementary Material, further inquiries can be directed to the corresponding author/s.

## Ethics Statement

The animal study was reviewed and approved by the Institutional Animal Care and Use Committee of the Fourth Military Medical University.

## Author Contributions

SX: designing the experiments, conducting the experiments, acquiring and analyzing the data, and writing the manuscript. WL: conducting the immunofluorescence staining and analyzing the data. YS, TW, YZ, and QH: conducting the animal experiments. JD and CD: conducting the western blot analysis. ZH: conducting the immunohistochemical staining. ZY: concept of the study, designing the experiments, and writing and editing the manuscript. XM: concept of the study, designing experiments, providing reagents, and writing and editing the manuscript. All authors contributed to the article and approved the submitted version.

## Conflict of Interest

The authors declare that the research was conducted in the absence of any commercial or financial relationships that could be construed as a potential conflict of interest.

## Publisher’s Note

All claims expressed in this article are solely those of the authors and do not necessarily represent those of their affiliated organizations, or those of the publisher, the editors and the reviewers. Any product that may be evaluated in this article, or claim that may be made by its manufacturer, is not guaranteed or endorsed by the publisher.

## Supplementary Material

Supplementary Figure 1 Cell proliferation in the mechanically stretched skin. **(A)** Immunohistochemistry (IHC) staining of Ki67 in mechanically stretched skin. Proliferative cells were mainly located in the basal layer of the epidermis and near the dermal hair follicles. **(B)** More proliferating Ki67+ cells were present in both the epidermis and dermis of the metformin-treated skin.

Supplementary Figure 2 Raw data of Figure 3C.

Supplementary Figure 3 Raw data of Figure 5C.

Supplementary Figure 4 Raw data of Figure 5G.

Supplementary Figure 5 Raw data of Figure 5K.

Supplementary Figure 6 Raw data of Figure 8C.
