# Supplementary figures and images for: Metformin Promotes Mechanical Stretch-Induced Skin Regeneration by Improving the Proliferative Activity of Skin-Derived Stem Cells
Source: Front Med (Lausanne). 2022 May 24;9:813917. doi: 10.3389/fmed.2022.813917 (PMC9170926; doi:10.3389/fmed.2022.813917)

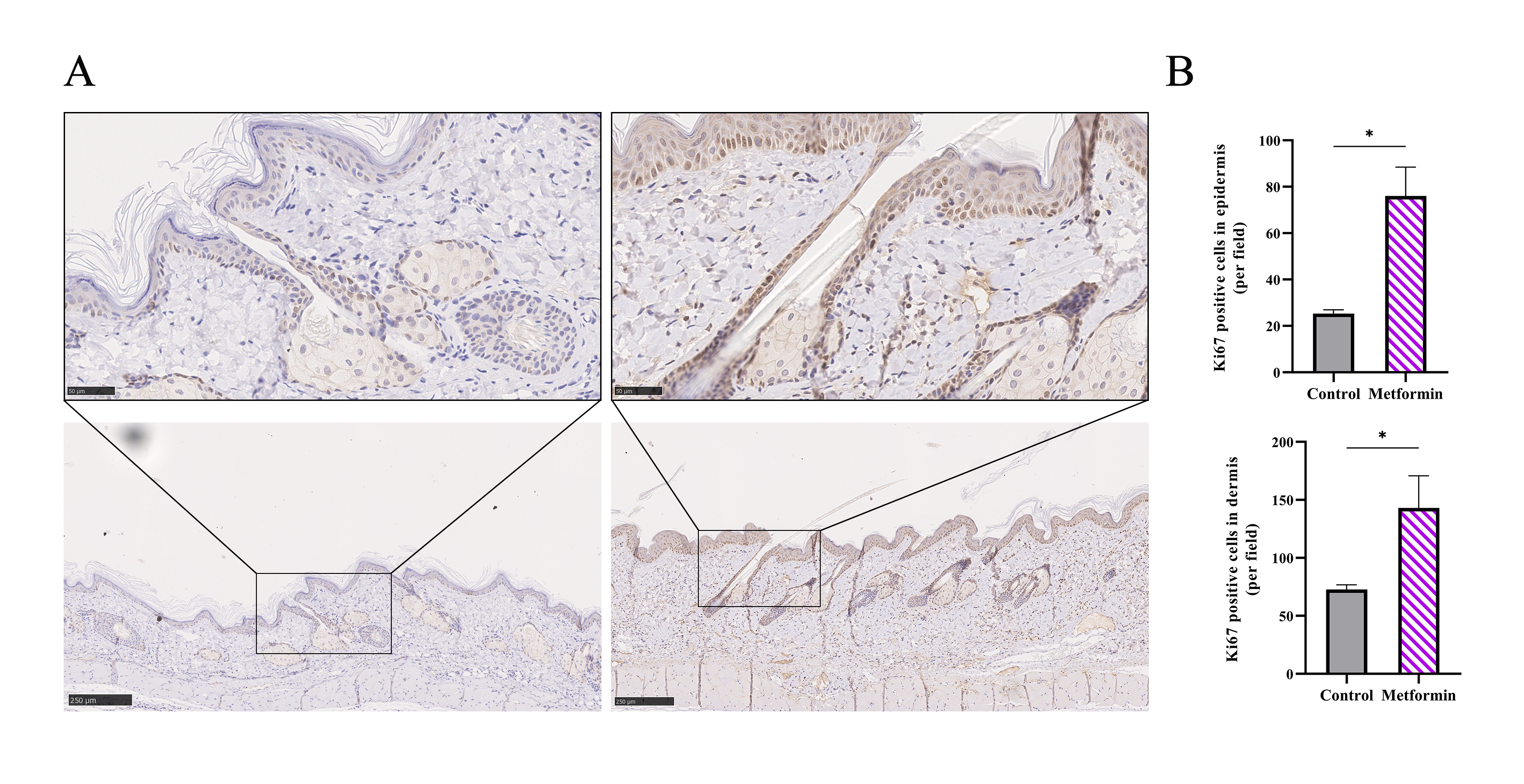

Supplement: Supplementary Figure 1 — Cell proliferation in the mechanically stretched skin. (A) Immunohistochemistry (IHC) staining of Ki67 in mechanically stretched skin. Proliferative cells were mainly located in the basal layer of the epidermis and near the dermal hair follicles. (B) More proliferating Ki67+ cells were present in both the epidermis and dermis of the metformin-treated skin. [file Image_1.JPEG]

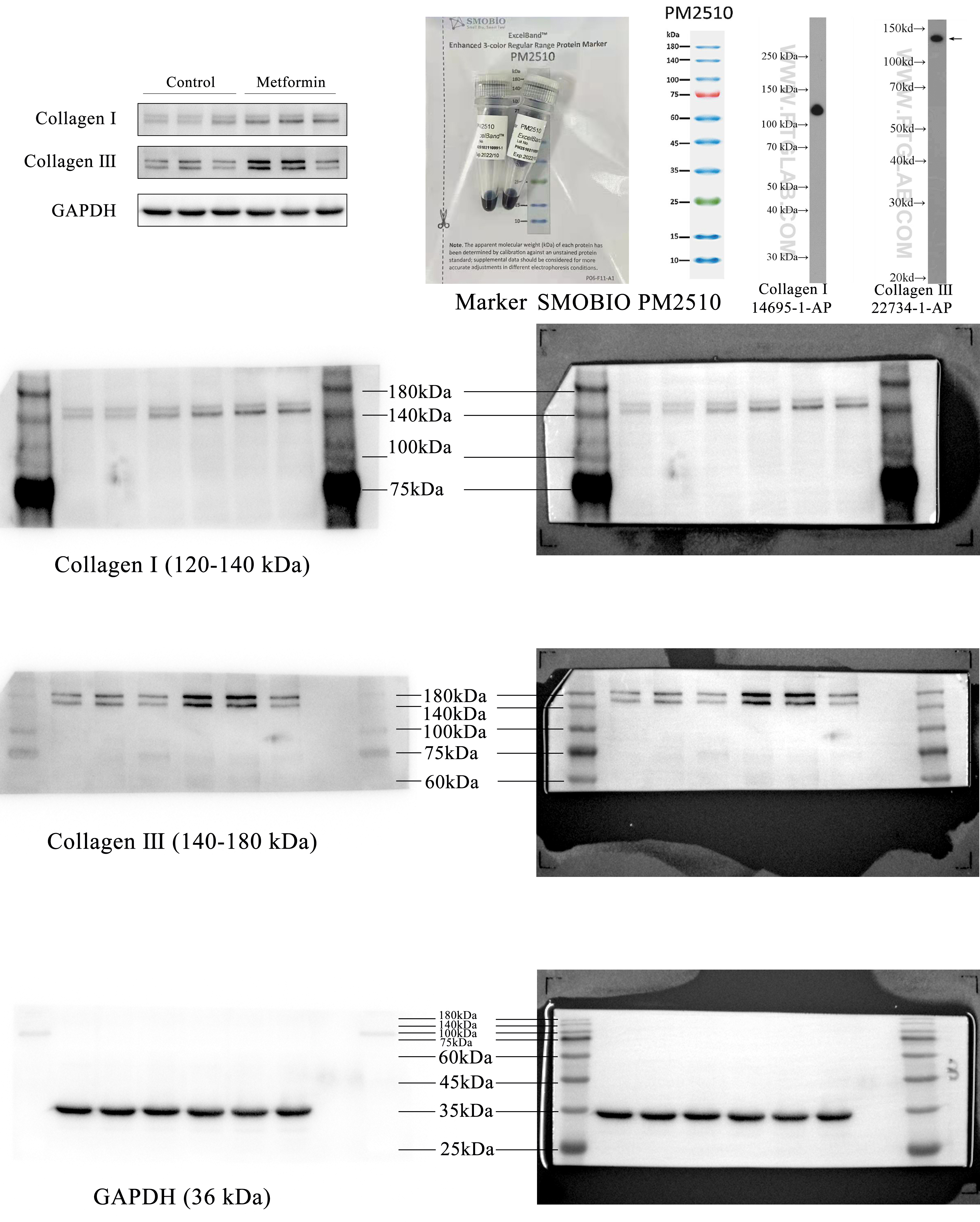

Supplement: Supplementary Figure 2 — Raw data of Figure 3C. [file Image_2.JPEG]

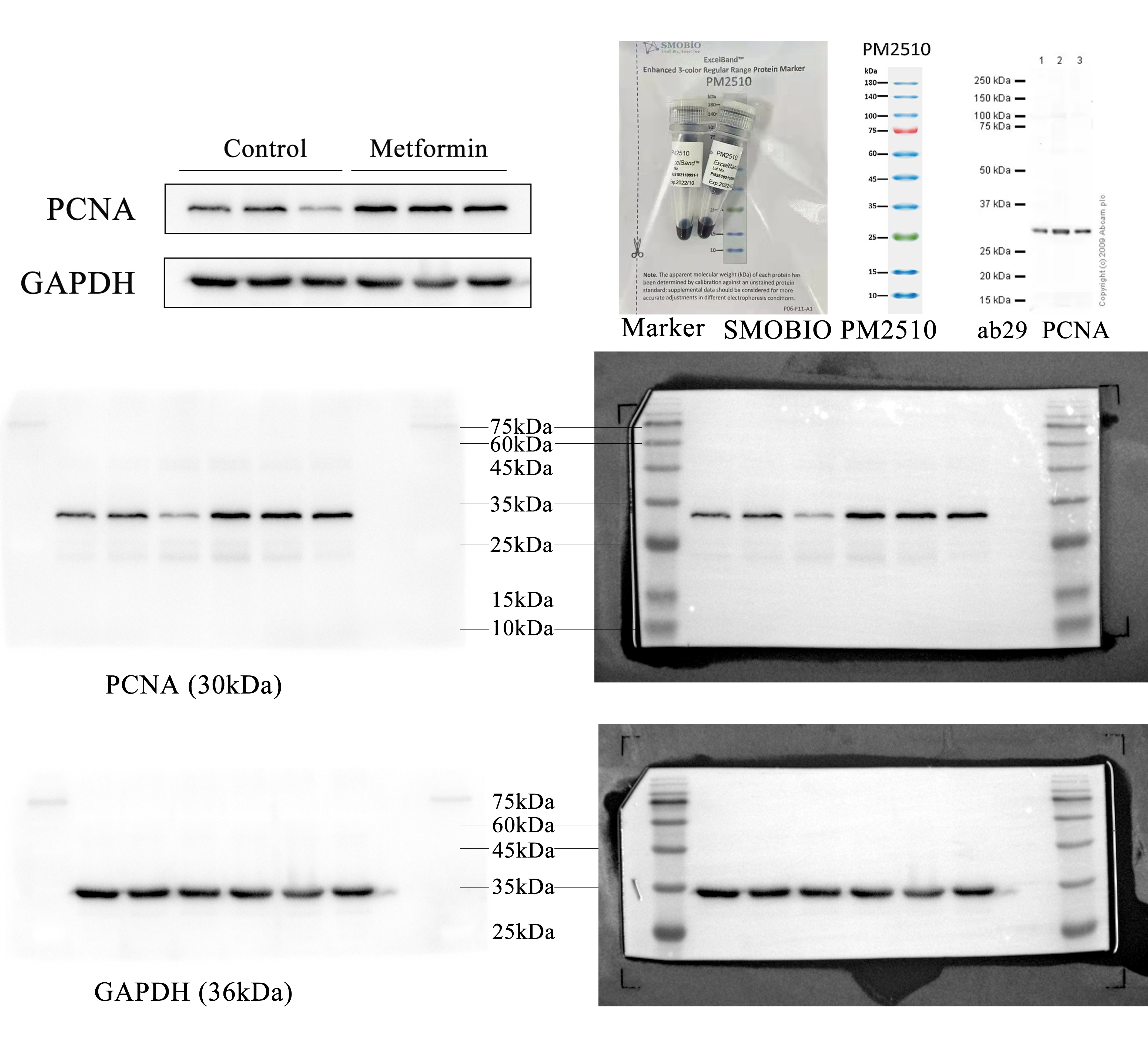

Supplement: Supplementary Figure 3 — Raw data of Figure 5C. [file Image_3.JPEG]

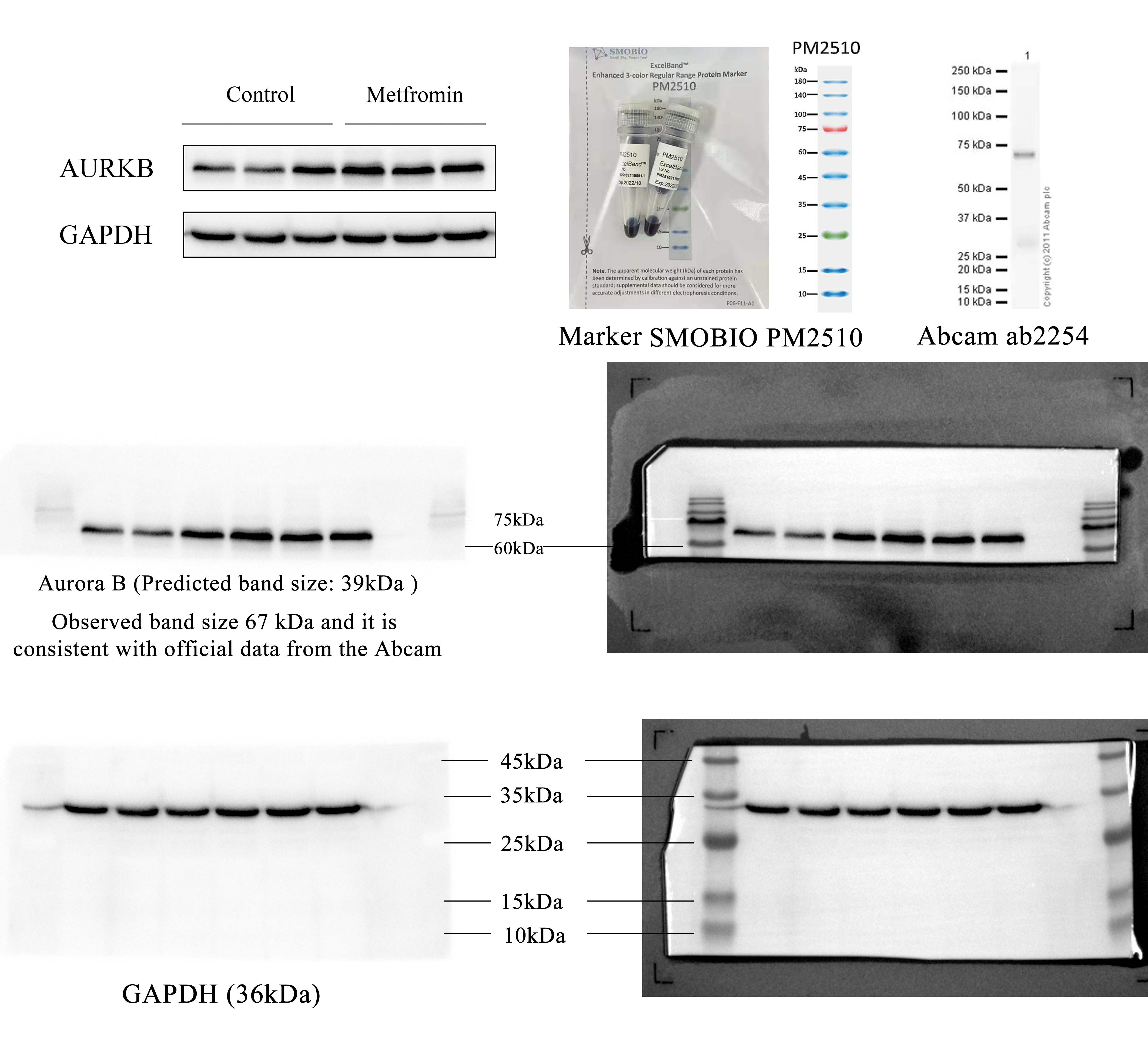

Supplement: Supplementary Figure 4 — Raw data of Figure 5G. [file Image_4.JPEG]

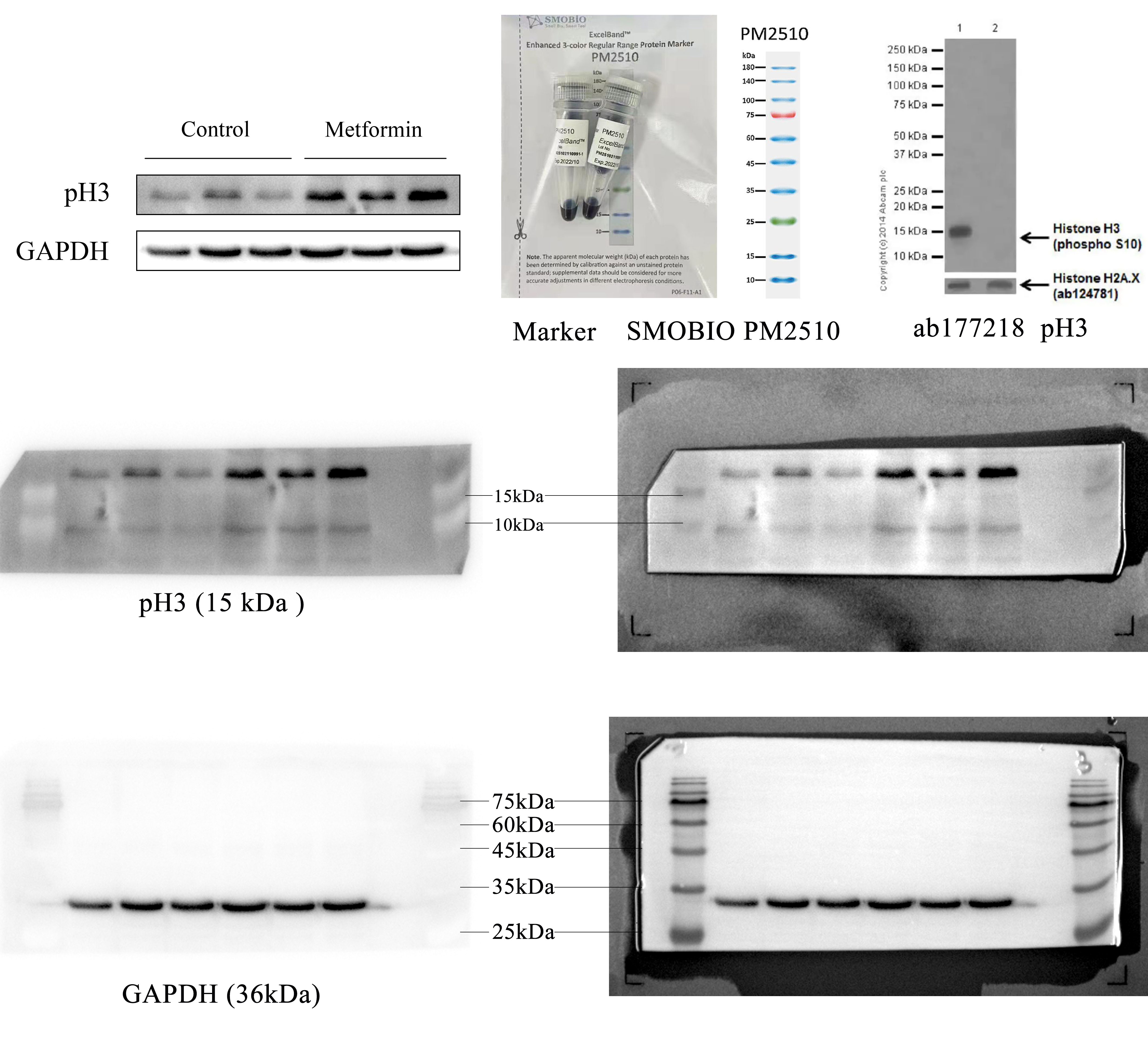

Supplement: Supplementary Figure 5 — Raw data of Figure 5K. [file Image_5.JPEG]

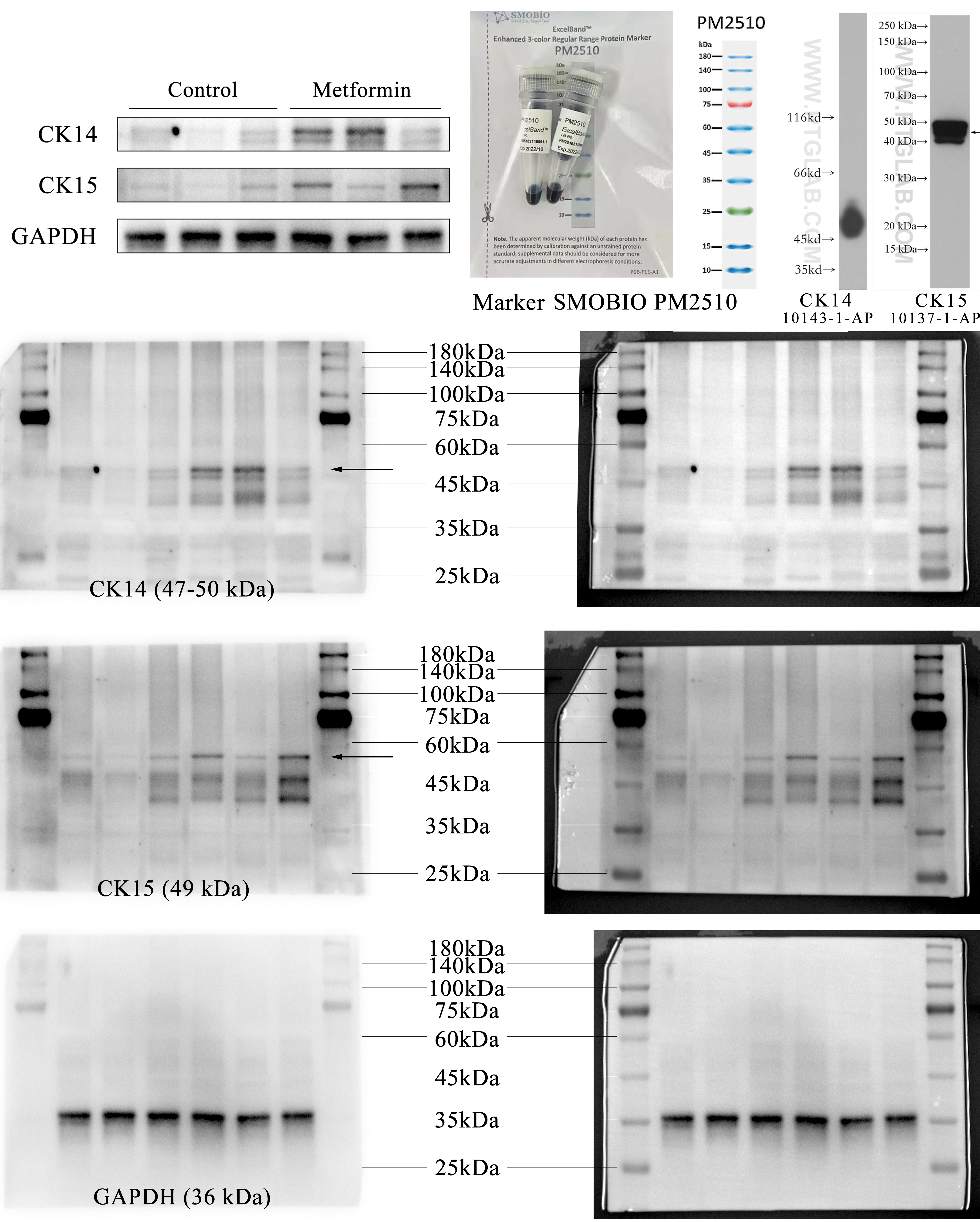

Supplement: Supplementary Figure 6 — Raw data of Figure 8C. [file Image_6.JPEG]
